# Effects of peripheral blood neutrophil/lymphocyte ratio levels and their changes on the prognosis of patients with early cervical cancer

**DOI:** 10.3389/fonc.2023.1139809

**Published:** 2023-09-27

**Authors:** Jun-Qiang Du, Fan Zhang, Chao-Qun Wang, Ju-Fan Zhu, Li-Xia Xu, Yi-Heng Yang, Meng-Fei Han, Yan Hu

**Affiliations:** ^1^ Department of Gynecology, Affiliated Dongyang Hospital of Wenzhou Medical University, Dongyang, Zhejiang, China; ^2^ Department of Pathology, Affiliated Dongyang Hospital of Wenzhou Medical University, Dongyang, Zhejiang, China; ^3^ Department of Gynecology, The First Affiliated Hospital of Wenzhou Medical University, Wenzhou, Zhejiang, China

**Keywords:** cervical cancer, neutrophil/lymphocyte ratio, prognosis, NLR, peripheral blood

## Abstract

Although some studies have reported on the levels and clinical significance of peripheral blood neutrophil/lymphocyte ratio (NLR) in cervical cancer, the role of NLR levels and their changes preoperatively and postoperatively in early cervical cancer remain unclear. Our analyses explored the preoperative and postoperative NLR in 203 patients with stage I–IIA cervical cancer and evaluated the relationship between NLR changes, clinicopathological characteristics, and patient prognosis. The cut-off preoperative and postoperative NLR values were determined using receiver operating characteristic curve analysis. Preoperative NLR correlated with age, menopausal status, tumor size, and vascular infiltration, whereas postoperative NLR correlated with tumor differentiation. Patients with cervical cancer with a high preoperative NLR had significantly shorter overall survival (OS) and progression-free survival (PFS) than other patients, whereas PFS was significantly lower in the high postoperative NLR group. When comparing postoperative and preoperative NLR values, we observed a significantly higher rate of increase in postmenopausal patients and those without vascular infiltration than that among premenopausal patients and those with vascular infiltration. However, no clear difference in prognosis was observed between the groups with increased and decreased NLR. Therefore, a high peripheral blood NLR may predict a poor prognosis in patients with early cervical cancer. The effect of NLR changes on the prognosis of patients with cervical cancer requires further verification in multicenter studies.

## 1 Introduction

Cervical cancer is one of the most common malignancies in women worldwide, and is associated with the highest mortality rate among all gynecological cancers. Recently, advances have been made in surgical approaches and radiotherapy. Despite the emergence of integrated treatment systems, such as targeted and gene therapies, cervical cancer remains one of the major causes of death in middle-aged and older women, with the age of onset tending to decrease over time (1). According to data obtained from the National Cancer Center in 2018, the five-year survival rate for cervical cancer in China was 59.8%, which is significantly lower than that in western countries (2). Improvements in the prevention and treatment of cervical cancer are urgently needed.

Recently, additional peripheral blood indicators, including changes in the number of neutrophils, lymphocytes, and other inflammatory cells, have been associated with tumorigenesis and prognosis (3). Previous studies have demonstrated that the peripheral blood neutrophil-to-lymphocyte ratio (NLR) reflects the balance between the body’s tumor inflammatory response and anti-tumor immunity (4, 5). Furthermore, the NLR is closely related to the prognosis of many malignancies, such as gastric cancer, colon cancer, primary hepatocellular carcinoma, and esophageal cancer (6–9). NLR, was first proposed by Bass et al. in 1983 (10), and it is the ratio of absolute neutrophil and lymphocyte values. An increase or decrease in the NLR is closely associated with patient prognosis; however, its prognostic value in early cervical cancer remains unclear. Although a few studies have reported on the clinical prognostic value of preoperative NLR in cervical cancer, controversy remains. We reviewed the literature to identify relevant reports regarding whether the postoperative NLR and its changes relative to the preoperative NLR affect patient prognosis, but there are few relevant reports on this matter. Therefore, further investigating the value and prognostic significance of preoperative NLR, postoperative NLR, and associated changes in cervical cancer preoperatively and postoperatively is of great clinical significance. If a simple clinical index is determined to predict patient prognosis preoperatively or during treatment, it can be used to guide and stratify the clinical management of different patients and improve patient prognosis.

## 2 Materials and methods

### 2.1 Patients and samples

Clinical, pathological, and prognostic data were collected from 203 patients with stage I–IIA cervical cancer who were initially treated at our hospital between January 2012 and December 2017. The case inclusion criteria were as follows: (i) with pathologically confirmed squamous, adenocarcinoma, or adenosquamous carcinoma of the cervix; (ii) met the clinical staging criteria of the International Federation of Gynecology and Obstetrics (FIGO, 2009), and had their staging determined by more than two chief physicians with extensive experience in gynecologic tumors, including stage I–IIA tumors; (iii) had undergone initial radical cervical cancer surgery in our hospital and did not receive chemotherapy or other antineoplastic treatment preoperatively; (iv) with complete peripheral blood test results available 7 days preoperatively and 20–40 days postoperatively; (v) no preoperative combined autoimmune diseases, hematologic diseases, or acute or chronic infections; and (vi) with complete clinical, pathological, and follow-up data. The exclusion criteria were as follows: (i) did not receive surgical treatment; (ii) incomplete case information or follow-up data; (iii) received neoadjuvant chemotherapy or hormone therapy preoperatively; and (iv) preoperative autoimmune, hematologic diseases, or acute or chronic infections. This study was approved by the Medical Ethics Committee of Dongyang Hospital, Wenzhou Medical University (approval no.: Dongrenyi 2022-YX-226).

### 2.2 Data collection

Data on clinical factors relevant to the prognosis of patients with cervical cancer were also collected. These items included age at diagnosis, menopausal status, clinical stage, type of pathology, degree of histological differentiation, tumor size, degree of stromal invasion, vaginal wall involvement, parametrial involvement, endometrial involvement, lymph node metastasis, vascular invasion, time to recurrence, survival time, and peripheral blood results within 7 days preoperatively and 20–40 days postoperatively but before any chemotherapy or chemoradiation. The NLR was calculated by dividing the absolute neutrophil count by the absolute lymphocyte count.

### 2.3 Treatment

Altogether, 203 patients who had received radical surgery for early stage cervical cancer were included in this study. Of these patients, 183 underwent open surgery and 20 underwent laparoscopic surgery. Patients aged <45 years had the opportunity to preserve their ovaries if they wished, and postoperative follow-up supplemental radiotherapy was performed according to the NCCN guidelines.

### 2.4 Patient follow-up

Follow-up began on postoperative day 1and was conducted mainly through outpatient follow-up, telephone follow-up, and patient readmission records until April 31, 2022. Progression-free survival (PFS) was defined as the time from postoperative day l to the time when the patient experienced tumor relapse/metastasis. Overall survival (OS) was defined as the time from postoperative day l to the time of death (excluding deaths due to non-tumor factors).

### 2.5 Statistical analysis

The SPSS 19.0 statistical software (SPSS Inc., Chicago, IL, USA) was used to analyze the data. Categorical data are expressed as composition ratios or rates, and the χ (2) test was used to compare the differences in the NLR between cohorts. The measurement data are expressed as means ± standard deviations (x ± S). Survival analysis was performed using the Kaplan–Meier method and the log-rank test to compare the differences in survival curves between cohorts. Multifactorial survival analysis was performed using the Cox proportional hazards model to identify the independent risk factors affecting the prognosis of patients with cervical cancer. Significance was set at *P*<0.05.

## 3 Results

### 3.1 Characteristics and distribution of the study population

Altogether, 203 patients were included in this study after strict screening according to the inclusion criteria; their ages ranged from 24 to 76 years. The average patient age was 52.38 ± 10.05 years, 86 patients were <50 years old, and 117 were ≥50 years old. The follow-up period ranged from 5 to 132 months, with a median of 71.5 months. Among the study population, 102 were premenopausal and 101 were menopausal. According to the 2009 International Federation of Gynecology and Obstetrics Clinical Staging Criteria for Cervical Cancer, 148 patients had clinical stage I and 55 had clinical stage II cervical cancer. Regarding pathological types, 186 patients had squamous carcinomas, 12 had adenocarcinomas, and 5 had adenosquamous carcinomas. The degree of differentiation included 36 patients with highly differentiated (G1) carcinomas, 107 patients with medium differentiation (G2), 36 patients with low differentiation (G3), and 24 patients with unknown differentiation. The tumor size distribution was as follows:76 patients had tumors <2cm in diameter, 123 had tumors ≥2cm, and 4 patients had unknown tumor sizes. The depths of infiltration were <1/2 in 117 patients and ≥1/2 in 86 patients. The vaginal wall was not involved and was involved in 185 and 18 patients, respectively, and the parametrial tissue was not involved and was involved in 199 and 4 patients, respectively. The endometrium was not involved and was involved in 198 and 5 patients, respectively, and the lymph nodes were negative and positive for lymph node metastasis in 170 and 33 patients, respectively. Finally, 47 patients vascular invasion, whereas 156 patients demonstrated no vascular infiltration. Among 203 cervical cancer patients, 1 case had missing preoperative NLR data due to peripheral blood test loss, 2 cases had missing postoperative NLR data. Therefore, in this study, there were 202 cases with preoperative NLR, 201 cases with postoperative NLR, and 200 cases with changes in postoperative NLR relative to the preoperative NLR.

### 3.2 Determination of the NLR cut-off value

**Figure 1:**
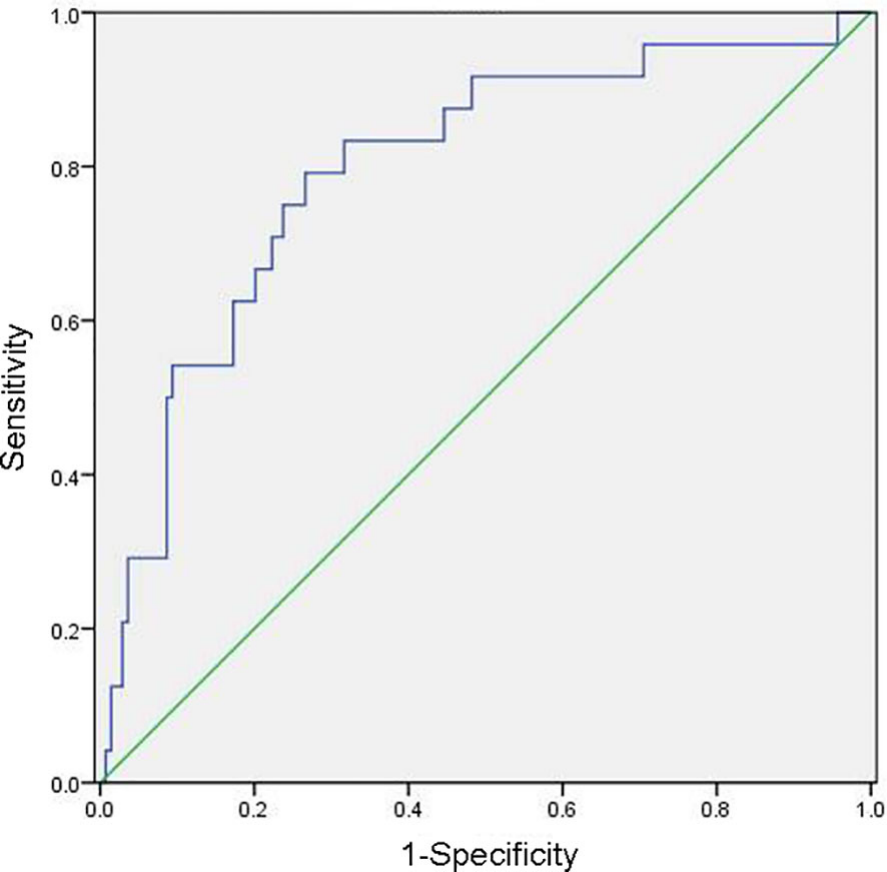
Receiver operating characteristic curve for determining the neutrophil/lymphocyte ratio cut-off value.

The mean preoperative NLR of the 202 patients with cervical cancer was 2.53, with a median of 2.20, while the mean postoperative NLR was 4.20, with a median of 2.64. The receiver operating characteristic curves of OS and PFS were plotted according to the NLR, and the cut-off value was selected with the maximum Youden index. The preoperative and postoperative NLR values were 3.75and 2.08, respectively, and these were determined as the evaluation cut-off values with sensitivity, specificity, and the area under the curve values of 79.17%, 73.38%, and 0.797, respectively (**Figure 1**). The high-level (30 patients) and low-level (172 patients) cohorts were identified by preoperative NLRs>3.75 and ≤3.75, respectively. Regarding postoperative levels, the high-level (126 patients) and low-level (76 patients) cohorts were identified by NLR >2.08 and ≤2.08, respectively.

### 3.3 Relationship between NLR levels and patient clinicopathological characteristics

Analysis of preoperative and postoperative NLR levels and changes in relation to various clinicopathological features revealed that preoperative NLR levels were significantly correlated with patient age (*P =* 0.013), menopause status (*P <*0.001), tumor size (*P* = 0.034), and vascular invasion (*P* = 0.015) (**Table 1**). Postoperative NLR was significantly correlated with the degree of tumor differentiation (*P* = 0.012) (**Table 1**). A comparison of the postoperative NLR with the preoperative NLR (with lower levels in one cohort and elevated levels in another) revealed that the proportion of patients with an elevated postoperative NLR was 65.7% (65/99) in the menopausal cohort and 51.5% (52/101) in the premenopausal cohort (*P =* 0.042). In the vascular infiltration cohort, the proportion of patients with an elevated postoperative NLR was 40.0% (18/45), which was significantly lower than in the nonvascular infiltration cohort (63.9%; 99/155; *P =* 0.004) (**Table 1**).

**Table 1:** Relationship between neutrophil/lymphocyte ratio (NLR) levels and changes and patient clinicopathological characteristics.

Clinical factors	Pre-operative NLR		X²	P	Post-operative NLR		X²	P	NLR changes^†^		X²	P
	Low cohort	High cohort			Low cohort	High cohort			Low cohort	High cohort		
Age (years)												
<50	67 (77.9)	19 (22.1)	6.210	**0.013**	29 (34.1)	56 (65.9)	0.854	0.355	36 (42.4)	49 (57.6)	0.044	0.833
≥50	105 (90.5)	11 (9.5)			47 (40.5)	69 (59.5)			>47 (40.9)	68 (59.1)		
Menopausal status												
Premenopausal	78 (76.5)	24 (23.5)	12.270	**0.000**	37 (36.6)	64 (63.4)	0.120	0.729	49 (48.5)	52 (51.5)	4.136	**0.042**
Postmenopausal	94 (94.0)	6 (6.0)			39 (39.0)	61 (61.0)			34 (34.3)	65 (65.7)		
Tumor stage												
I	127 (86.4)	20 (13.6)	0.663	0.416	56 (38.1)	91 (61.9)	0.019	0.891	62 (42.5)	84 (57.5)	0.208	0.649
II	45 (81.8)	10 (18.2)			20 (37.0)	34 (63.0)			21 (38.9)	33 (61.1)		
Tumor differentiation												
High	29 (80.5)	7 (19.5)	0.641	0.423	7 (20.0)	28 (80.0)	6.255	**0.012**	10 (28.6)	25 (71.4)	3.523	0.061
Moderate-poor	122 (85.9)	20 (14.1)			61 (43.0)	81 (57.0)			65 (46.1)	76 (53.9)		
Tumor size (cm)												
<2cm	70 (92.1)	6 (7.9)	4.498	**0.034**	30 (40.0)	45 (60.0)	0.307	0.580	27 (36.0)	48 (64.0)	1.167	0.280
≥2cm	99 (81.1)	23 (18.9)			44 (36.0)	78 (64.0)			53 (43.8)	68 (56.2)		
Depth of invasion												
<1/2	103 (88.0)	14 (12.0)	1.831	0.176	44 (38.3)	71 (61.7)	0.023	0.879	46 (40.0)	69 (60.0)	0.251	0.617
≥1/2	69 (81.1)	16 (18.9)			32 (37.2)	54 (62.8)			37 (43.5)	48 (56.5)		
Involvement of the vaginal wall												
No	158 (85.9)	26 (14.1)	0.330	0.566	69 (37.7)	114 (62.3)	0.010	0.921	74 (40.7)	108 (59.3)	0.589	0.443
Yes	14 (77.8)	4 (22.2)			7 (38.9)	11 (61.1)			9 (50.0)	9 (50.0)		
Parietal involvement (of the uterus)												
No	169 (85.4)	29 (14.6)		0.477	74 (37.6)	123 (62.4)	0.000	1.000	81 (41.3)	115 (58.7)	0.000	1.000
Yes	3 (75.0)	1 (25.0)			2 (50.0)	2 (50.5)			2 (50.0)	2 (50.0)		
Endothelial involvement												
No	168 (85.3)	29 (14.7)		0.556	76 (38.8)	120 (61.2)	1.687	0.194	83 (42.6)	112 (57.4)	2.096	0.148
Yes	4 (80.0)	1 (20.0)			0 (0.0)	5 (100.0)			0 (0.0)	5 (100.0)		
Lymph node metastasis												
No	147 (86.5)	23 (13.5)	0.897	0.344	67 (39.9)	101 (60.1)	1.865	0.172	71 (42.3)	97 (57.7)	0.251	0.616
Yes	25 (78.1)	7 (21.9)			9 (27.3)	24 (72.7)			12 (37.5)	20 (62.5)		
Vascular infiltration												
Nos	138 (88.5)	18 (11.5)	5.946	**0.015**	58 (37.4)	97 (62.6)	0.044	0.834	56 (36.1)	99 (63.9)	8.186	**0.004**
Yes	34 (73.9)	12 (26.1)			18 (39.1)	28 (61.9)			27 (60.0)	18 (40.0)		

^†^The postoperative NLR was divided into lower and elevated cohorts relative to the preoperative NLR.

^†^The bold values represent P-values that are all less than 0.05. The significance level was set at P<0.05.

### 3.4 Relationship between NLR levels and prognosis of patients with cervical cancer

The Kaplan–Meier method was used to depict the 5-year OS and PFS curves of the patients, and the log-rank test was used to compare survival differences between the cohorts. Survival analysis revealed that among the 165 patients with cervical cancer for whom follow-up information was obtained, the 5-year OS and PFS rates were 84.8% (140/165) and 79.4% (131/165), respectively.

**Figure 2:**
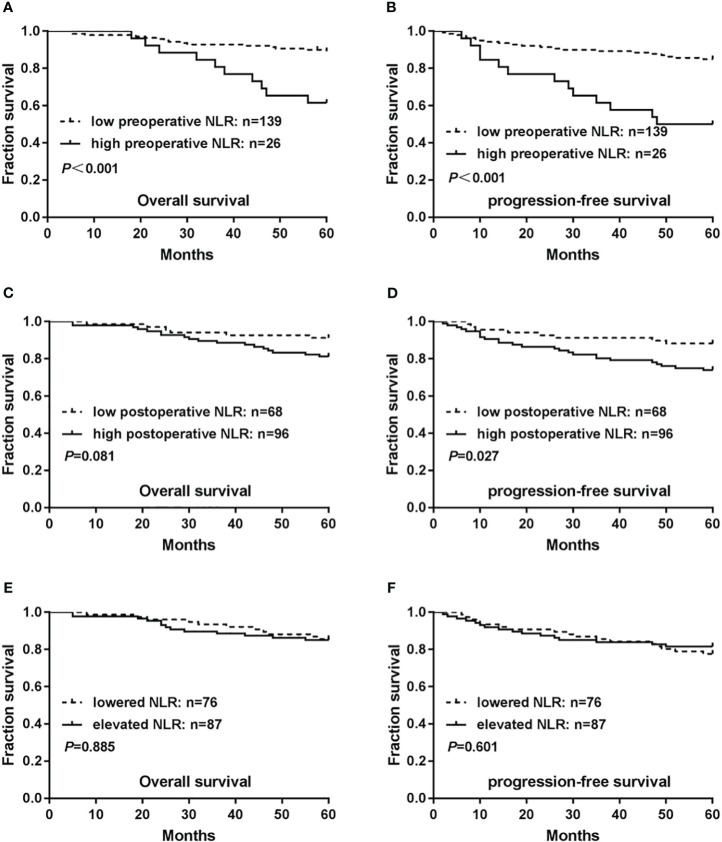
Relationship between neutrophil/lymphocyte ratio (NLR) levels and prognosis of patients with cervical cancer. **(A)** Associations of preoperative NLR levels with overall survival(OS). **(B)** Associations of preoperative NLR levels with progression-free survival (PFS). **(C)** Associations of postoperative NLR levels with OS. **(D)** Associations of postoperative NLR levels with PFS. **(E)** Associations of NLR changes preoperatively and postoperatively with OS. **(F)** Associations of NLR changes preoperatively and postoperatively with PFS. *P*-values were calculated using the Mantel–Cox log-rank test.

The 5-year mean survival time was 50.85 months, and the OS rate was 61.5% (16/26) for patients in the high-level preoperative NLR cohort. These values were significantly lower than those in the low-level preoperative NLR cohort [5-year mean survival time of 56.72 months and OS rate of 89.2% (124/139) (*P* < 0.001)] (**Figure 2A**). The 5-year mean PFS time and rate were 42.19 months and 50.0% (13/26), respectively, for patients in the high-level preoperative NLR cohort, which was significantly lower than that in the low-level preoperative NLR cohort [5-year mean PFS time and rate were 54.63 months and 84.9% (118/139), respectively (*P* < 0.001)] (**Figure 2B**).

Both the 5-year mean survival time of 54.82 months and the OS rate of 81.3% (78/96) were lower in patients in the high-level postoperative NLR cohort than in those in the low-level postoperative NLR cohort [5-year mean survival time of 57.27 months and OS rate of 91.2% (62/68)]. However, this difference was not statistically significant (*P*=0.081) (**Figure 2C**). The 5-year mean PFS time was 50.65 months and the PFS rate was 74.0% (71/96), which were significantly lower in the high-level postoperative NLR cohort than in the low-level postoperative NLR cohort [5-year mean PFS time of 55.68 months and PFS rate of 88.2% (60/68) (*P=*0.027)](**Figure 2D**).

The postoperative NLR was divided into lower and elevated cohorts relative to the preoperative NLR. A comparison of the two cohorts revealed that the 5-year mean survival time in the lower cohort was 56.55 months, and the OS rate was 85.5% (65/76).The 5-year mean survival time in the elevated cohort was 55.16 months, and the OS rate was 85.1% (74/87). However, these differences were not statistically significant (*P*= 0.885) (**Figure 2E**). The 5-year mean PFS time was 52.93 months, and the PFS rate was 77.6% (59/76) in the postoperative lowered cohort. The 5-year mean PFS time was 52.47 months, and the PFS rate was 81.6% (71/87) in the postoperative elevated cohort, without statistical significance(*P*=0.601) (**Figure 2F**).

Multifactorial analysis revealed high-levels of preoperative NLR (hazard ratio [HR]=3.511; 95% confidence interval [CI] = 1.546–7.977; *P*=0.003), and lymph node metastasis (HR=3.562; 95% CI = 1.568–8.092; *P*=0.002) as independent influencing factors on OS in patients with cervical cancer. High preoperative NLR (HR=3.552; 95% CI = 1.701-7421; *P*=0.001), clinical stage II (HR=2.676; 95% CI = 1.297–5.523; *P*=0.008), and lymph node metastasis (HR=2.798; 95% CI = 1.324–5.912; *P*=0.007) independently influenced PFS in patients with cervical cancer.

## 4 Discussion

Previous studies have reported that inflammatory responses play an important role in tumorigenesis, development, invasion, and metastasis (11–15). The main manifestation in the peripheral blood is an alteration in the number of inflammatory cells such as neutrophils and lymphocytes. The biological roles of neutrophils and lymphocytes in cancer development and prognosis include the following aspects (11, 12). First, neutrophils play a key role in coordinating innate and adaptive immune responses by releasing cytokines, chemokines, and antigens. Second, neutrophil-produced mediators and inflammatory factors promote the formation of a microenvironment favorable for tumor cell growth. Third, neutrophil-produced metalloproteinases promote the involvement of vascular endothelial growth factor in angiogenesis, leading to tumor angiogenesis and distant metastasis. Fourth, neutrophils also participate in the induction of tumor suppressor gene mutations; degradation of immunoglobulins, receptors, and complements; and promotion of tumor cell proliferation and differentiation. Fifth, lymphocytes play a crucial role in suppressing tumor growth and promoting tumor cell apoptosis during the cellular immune processes of tumors. A decrease in lymphocytes reduces the immune defense function against tumors and the ability to destroy tumor cells in the body, thereby promoting tumor occurrence and progression. Sixth, circulating tumor cells may also occur even in early stages of cancer and these are often associated with clusters of neutrophils. Such circulating tumor cells and clusters are likely to be ‘counted’ as neutrophils in the hematological coulter counter analyses. In recent years, peripheral blood NLR has become a research hotspot because it has demonstrated better prognostic predictive value in various tumors, such as renal cell carcinoma (16), intrahepatic cholangiocarcinoma (17), esophageal cancer (18), and colorectal cancer (19). Although a few studies have reported on the clinical prognostic value of preoperative NLR in cervical cancer, its clinical and cutoff values remain controversial (20–25).

The NCCN guidelines of 2015 included tumor size, vascular infiltration, and degree of differentiation as intermediate risk factors for cervical cancer. These poor prognosis risk factors guide decisions regarding whether to supplement surgery with chemoradiotherapy. Our data demonstrate that preoperative and postoperative NLR may also be a prognostic indicator in patients with early cervical cancer.

The PFS and OS data demonstrate that patients with a high preoperative NLR have a worse prognosis. This is consistent with other published studies by Li et al. (21), Wu et al. (23) and Ethier et al. (26). In a meta-analysis of 6041 patients with cervical cancer, Zou et al. (27) have reported that the median cut-off value for NLR was 2.46 and higher pre-treatment peripheral blood NLR levels were associated with poorer OS and shorter PFS. However, a predefined cut off value has not been suggested and will require further multicenter, international, large-scale studies.

This study evaluated for the first time that there are no appreciable differences in outcome for patients whose NLR changes significantly pre and post operatively. However, owing to the small sample size of this study, as well as the geographical and population differences, our results may have limited generalizability and need to be followed up with a multicenter and large-scale study.

In conclusion, high NLR has some prognostic value in patients with early stage cervical cancer, and peripheral blood could be used as an auxiliary indicator of tumor prognosis owing to the convenience and reproducibility of the test. However, no uniform standard exists regarding the cutoff NLR value, and the findings of this single-center study require supporting data from future multicenter, large-sample studies.

## Data availability statement

The original contributions presented in the study are included in the article/supplementary material. Further inquiries can be directed to the corresponding authors.

## Ethics statement

The studies involving humans were approved by Medical Ethics Committee of Dongyang Hospital, Wenzhou Medical University. The studies were conducted in accordance with the local legislation and institutional requirements. Written informed consent for participation was not required from the participants or the participants’ legal guardians/next of kin in accordance with the national legislation and institutional requirements.

## Author contributions

Conceptualization: J-QD, FZ, YH. Data curation: FZ, J-FZ, L-XX. Funding acquisition: FZ, YH. Methodology: J-QD, FZ, C-QW, Y-HY, M-FH. Resources: J-QD, FZ. Writing – original draft: J-QD, FZ. Writing – review & editing: C-QW, YH. All authors contributed to the article and approved the submitted version.
